# A 4-cm lipoma of the transverse colon causing colonic intussusception: A case report and literature review

**DOI:** 10.3892/ol.2014.2278

**Published:** 2014-06-24

**Authors:** XIAO-CONG ZHOU, KE-QIONG HU, YI JIANG

**Affiliations:** 1 Department of Surgery, The Dingli Clinical Institute of Wenzhou Medical University (Wenzhou Central Hospital), Wenzhou, Zhejiang 325000, P.R. China; 2 Department of Clinical Pharmacy, The Dingli Clinical Institute of Wenzhou Medical University (Wenzhou Central Hospital), Wenzhou, Zhejiang 325000, P.R. China; 3 Department of Pathology, The Dingli Clinical Institute of Wenzhou Medical University (Wenzhou Central Hospital), Wenzhou, Zhejiang 325000, P.R. China

**Keywords:** lipoma, colon, intussusception, diagnosis

## Abstract

Colonic lipomas are rare benign tumors. Colonic intussusception is an uncommon complication of colonic lipoma. The current study presents an unusual case of a 4-cm symptomatic lipoma of the transverse colon causing colonic intussusception. A 65-year-old female was admitted to Wenzhou Central Hospital (Wenzhou, Zhejiang, China) with intermittent pain in the left abdomen that had been present for two weeks. Colonoscopy revealed a 4×5-cm intraluminal spherical mass with erosional mucosa 60 cm above the anal verge, indicating the presence of a malignant gastrointestinal stromal tumor. Contrast-enhanced computed tomography revealed a well-defined fatty tissue mass of 4 cm in diameter in the distal transverse colon proximal to the splenic flexure, with intussusception. The patient underwent segmental resection of the transverse colon and intraoperative frozen sections were obtained. The intraoperative frozen sections revealed a submucosal lipoma of the transverse colon and thus, a conclusive diagnosis was achieved. The patient was followed up for one year and 10 months following the segmental resection of the transverse colon, with a good prognosis. This study may increase clinical awareness with regard to colonic lipomas. Furthermore, open surgery combined with use of intraoperative frozen sections should be recommended for large symptomatic colonic lipomas accompanied by colonic intussusception, thus avoiding unnecessary radical resection and improving patient prognosis.

## Introduction

Lipomas of the gastrointestinal tract are benign tumours and were first reported by Bauer in 1757 (1). Although colonic lipomas are the most common type of non-epithelial (mesenchymal) neoplasm of the gastrointestinal tract, they are rare benign tumors (2). Generally, colonic lipomas are asymptomatic and thus, they are usually detected incidentally during colonoscopy, surgery or autopsy (3). However, 25% of colonic lipomas are known to develop symptoms, particularly when their diameter is >2 cm (4). Symptoms include anemia, abdominal pain, constipation, diarrhea, bleeding and intussusception (5). The term ‘giant lipoma’ has been defined as a mass of >5 cm in diameter (6). Large colonic lipomas are often misdiagnosed as more serious pathology due to their rarity and variable presentation (3). Imaging modality, including computed tomography, contributes to the preoperative diagnosis of colonic lipomas as its imaging characteristics are relatively typical for adipose tissue. A firm diagnosis of colonic lipoma can be established fundamentally based on the histopathological examination. In 90% of cases, lipomas of the colon are localized at the submucous level, with only a few cases presenting in the subserosal layer (7). Although intussusception is a common disease in children, intussusception caused by colonic lipoma in adults is a rare condition, and is caused usually by a large pedunculated lipoma (8). The current study presents a rare case of a 4-cm sessile lipoma of the transverse colon causing colonic intussusception.

## Case report

**Figure 1:**
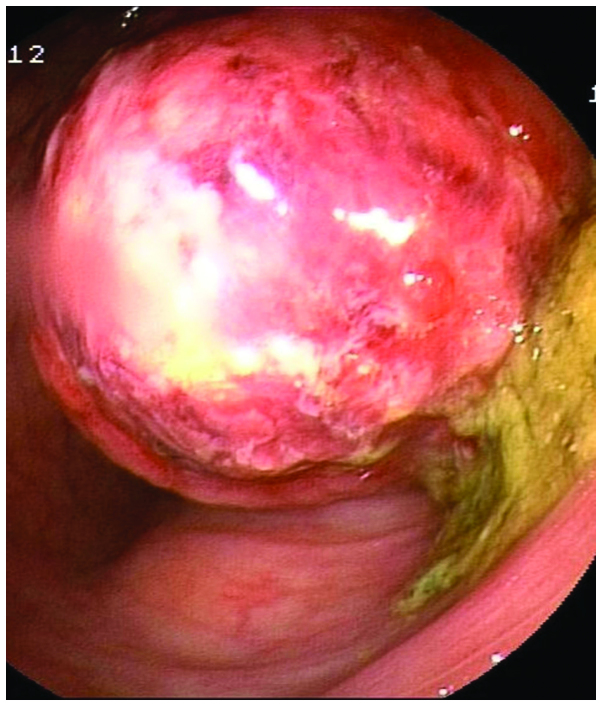
Colonoscopy image revealing a 4×5-cm intraluminal spherical mass, 60 cm above the anal verge, which prevented further progression of the endoscope. The mass was covered by a 2×3-cm superficial mucosal erosion, indicating the presence of a malignant gastrointestinal stromal tumor.

**Figure 2:**
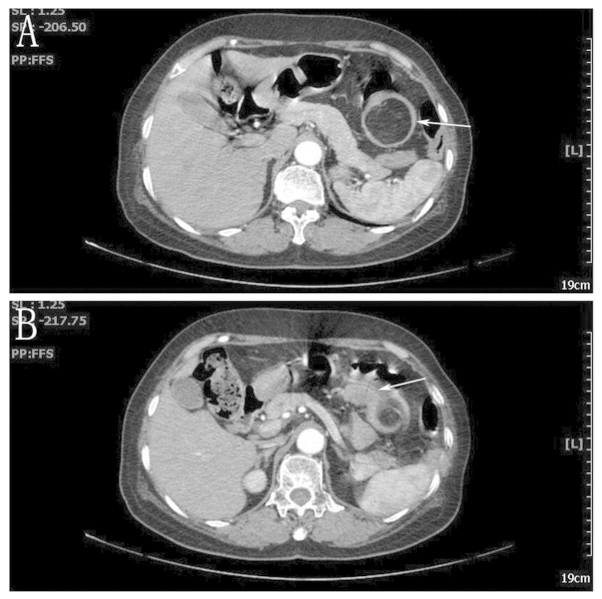

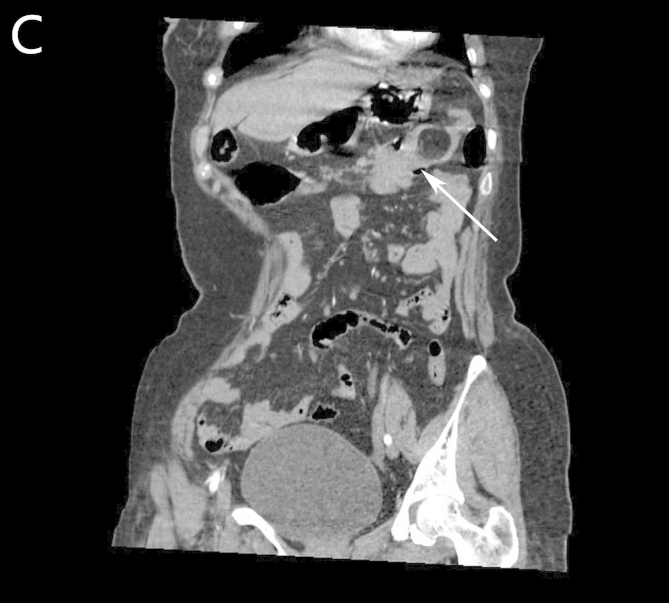
Contrast-enhanced computed tomography revealing (A) a well-defined fatty tissue mass of 4 cm in diameter (arrow) in the distal transverse colon proximal to the splenic flexure, with (B and C) intussusception (arrow) and local bowel-wall thickening.

In March 2012, a 65-year-old female was admitted to Wenzhou Central Hospital (Wenzhou, Zhejiang, China) with intermittent pain in the left abdomen that had been present for two weeks. The patient had no past history of cancer and no family history of colorectal cancer. The physical examination was unremarkable. The laboratory results revealed a white blood cell count of 12.5×10^9^ cells/l, a neutrophil count of 9.2×10^9^ cells/l (73.2%) and a C-reactive protein level of 10.4 mg/l. Tumor markers were within the normal ranges. Colonoscopy revealed a 4×5-cm intraluminal spherical mass, 60 cm above the anal verge, which prevented further progression of the endoscope. The mass was covered by a 2×3-cm superficial mucosal erosion, indicating the presence of a malignant gastrointestinal stromal tumor (Fig. 1). In addition, a biopsy of the mass revealed numerous ulcerative lesions with local epithelial regeneration. Furthermore, contrast-enhanced computed tomography (CT) revealed a well-defined fatty tissue mass of 4 cm in diameter in the distal transverse colon proximal to the splenic flexure (Fig. 2A), with intussusception (Fig. 2B and C) and local bowel-wall thickening.

**Figure 3:**
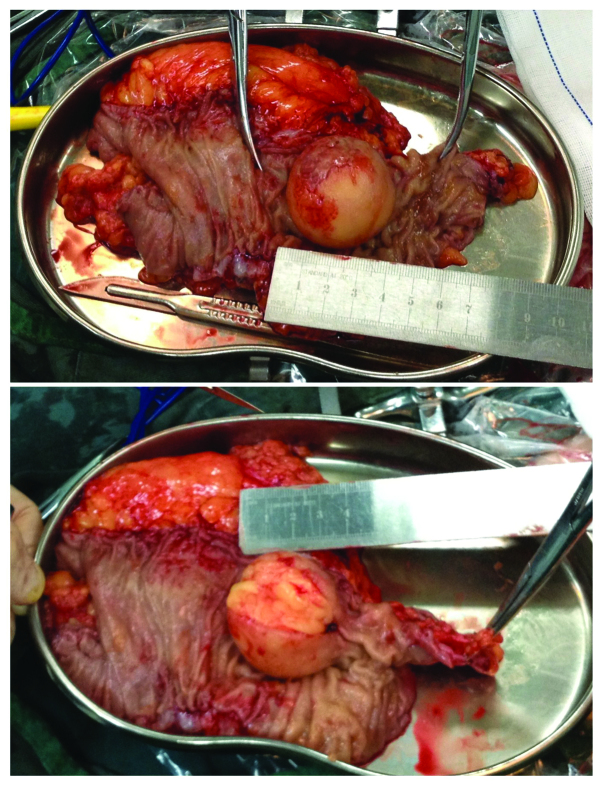
Macroscopic assessment of the resected specimen reveaing the presence of a yellow, round and broader-based 4×4-cm mass with the features of a lipoma.

**Figure 4:**
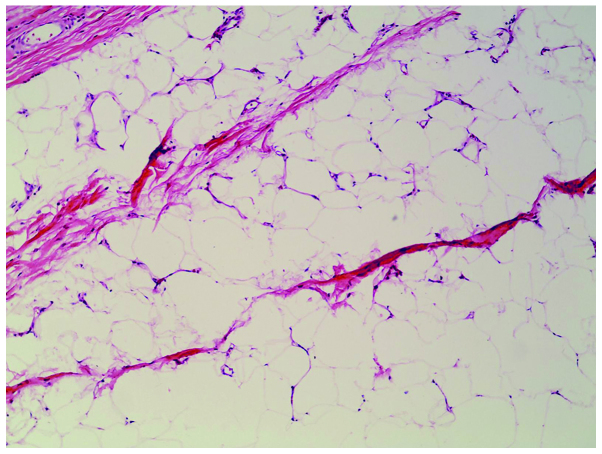
Histopathological examination of the resected specimen revealing a mass composed of mature fat cells, focal erosion and ulceration of the overlying colonic mucosa (hematoxylin and eosin stain; magnification, ×200).

**Figure 5:**
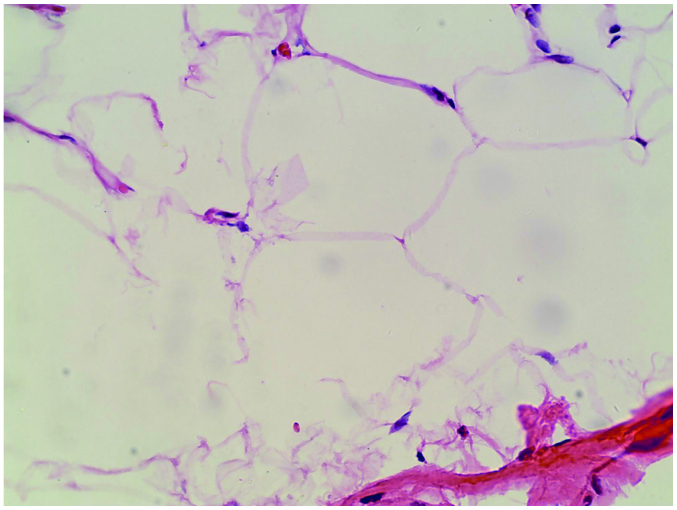
Histopathological examination of the resected specimen revealing a mass composed of mature fat cells, focal erosion and ulceration of the overlying colonic mucosa (hematoxylin and eosin stain; magnification, ×400).

The patient underwent segmental resection of the transverse colon following the initial diagnosis. The intraoperative frozen section revealed a submucosal lipoma of the transverse colon. No further resection was required. Macroscopic assessment of the resected specimen identified the presence of a yellow, round and broader-based 4×4-cm mass exhibiting the features of a lipoma (Fig. 3). Histopathological examination of the resected specimen revealed that the mass was composed of mature fat cells, focal erosion and ulceration of the overlying colonic mucosa (Figs. 4 and 5). A conclusive diagnosis of a submucosal lipoma of the transverse colon was achieved. The post-operative course was uneventful. The patient was followed up for one year and 10 months following the segmental resection of the transverse colon, with a good prognosis. Written informed consent was obtained from the patient for the publication of this case study and any accompanying images.

## Discussion

Colonic lipomas are rare benign tumors of the gastrointestinal tract and are classified as a type of benign non-epithelial tumor. The incidence of colonic lipoma ranges between 0.035 and 4.4% (9), and the peak incidence occurs within the fifth and sixth decades of life, most commonly in females (7,10). Usually, colonic lipoma is solitary, with the most common locations for solitary colonic lipoma being the ascending colon and cecum, followed by the transverse colon, descending colon, sigmoid colon and rectum (7). The majority of colonic lipomas are asymptomatic and do not require treatment, however, a small number may cause symptoms when the lesion is large, particularly those with a diameter >2 cm (11). Colonic intussusception is also a rare complication of colonic lipoma (12).

The size of colonic lipomas ranges between 2 mm and 30 cm and may mimic colonic malignancies (13). The present case revealed that large colonic lipomas and malignant tumors may be difficult to differentiate prior to resection if only endoscopic observations are used. Due to the non-specific clinical presentations and endoscopic appearance, including the multiple areas of erosion and ulceration that were identified on the mass surface, together with the relatively hard texture, the two may be indistinguishable. However, for colonic lipomas of a large size and in acutely ill patients, CT is the preferred diagnostic method, as the imaging characteristics of the tumors are fairly typical for adipose tissue (14).

However, an intraoperative frozen section may provide an accurate diagnosis to guide surgery (15). In the present case, the pre-operative biopsy during colonoscopy revealed numerous ulcerative lesions with local epithelial regeneration, without malignant tumor cells. The patient underwent segmental resection of the transverse colon and intraoperative frozen sections were obtained. As a result of the intraoperative frozen sections, which revealed a submucosal lipoma of the transverse colon, an unnecessary radical resection was avoided.

In conclusion, colonic lipoma is a relatively rare benign tumor, which as a clinical entity may be easily misdiagnosed as a malignant tumor. The clinical awareness of colonic lipomas must be increased. Open surgery combined with the use of intraoperative frozen sections should be recommended for large symptomatic colonic lipomas accompanied by colonic intussusception, thus avoiding unnecessary radical resection and improving the patient prognosis.
